# Role of immune-related endoplasmic reticulum stress genes in sepsis-induced cardiomyopathy: Novel insights from bioinformatics analysis

**DOI:** 10.1371/journal.pone.0315582

**Published:** 2024-12-13

**Authors:** Wan-Jing Zhen, Yan Zhang, Wei-Dong Fu, Xiao-Lei Fu, Xin Yan

**Affiliations:** 1 Department of Anesthesiology, the Second Affiliated Hospital of Fujian Medical University, Quanzhou, Fujian Province, China; 2 Department of Anesthesiology, Zhuzhou Central Hospital (Zhuzhou Hospital Affiliated to Xiangya School of Medicine), Zhuzhou, Hunan Province, China; 3 Department of Cardiovascular Medicine, Zhuzhou Central Hospital (Zhuzhou Hospital Affiliated to Xiangya School of Medicine), Zhuzhou, Hunan Province, China; Jhargram Raj College, INDIA

## Abstract

**Background:**

The current study aims to elucidate the key molecular mechanisms linked to endoplasmic reticulum stress (ERS) in the pathogenesis of sepsis-induced cardiomyopathy (SIC) and offer innovative therapeutic targets for SIC.

**Methods:**

The study downloaded dataset GSE79962 from the Gene Expression Omnibus database and acquired the ERS-related gene set from GeneCards. It utilized weighted gene co-expression network analysis (WGCNA) and conducted differential expression analysis to identify key modules and genes associated with SIC. The SIC hub genes were determined by the intersection of WGCNA-based hubs, DEGs, and ERS-related genes, followed by protein-protein interaction (PPI) network construction. Enrichment analyses, encompassing GO, KEGG, GSEA, and GSVA, were performed to elucidate potential biological pathways. The CIBERSORT algorithm was employed to analyze immune infiltration patterns. Diagnostic and prognostic models were developed to assess the clinical significance of hub genes in SIC. Additionally, in vivo experiments were conducted to validate the expression of hub genes.

**Results:**

Differential analysis revealed 1031 differentially expressed genes (DEGs), while WGCNA identified a hub module with 1327 key genes. Subsequently, 13 hub genes were pinpointed by intersecting with ERS-related genes. NOX4, PDHB, SCP2, ACTC1, DLAT, EDN1, and NSDHL emerged as hub ERS-related genes through the protein-protein interaction network, with their diagnostic values confirmed via ROC curves. Diagnostic models incorporating five genes (NOX4, PDHB, ACTC1, DLAT, NSDHL) were validated using the LASSO algorithm, highlighting only the prognostic significance of serum PDHB levels in predicting the survival of septic patients. Additionally, decreased PDHB mRNA and protein expression levels were observed in the cardiac tissue of septic mice compared to control mice.

**Conclusions:**

This study elucidated the interplay between metabolism and the immune microenvironment in SIC, providing fresh perspectives on the investigation of potential SIC pathogenesis. PDHB emerged as a significant biomarker of SIC, with implications on its progression through the regulation of ERS and metabolism.

## Introduction

Sepsis is a condition characterized by severe organ dysfunction following infection by various pathogenic microorganisms [1]. In 2017, an estimated 48.9 million septic patients were diagnosed worldwide, resulting in over 11 million deaths [2]. Recognizing its significant impact, the World Health Organization has identified sepsis as a global health priority [3]. Sepsis-induced cardiomyopathy (SIC), a cardiac impairment triggered by sepsis, is a prevalent complication marked by reduced left ventricular (LV) cardiac output [4, 5]. SIC is known to involve multiple pathophysiological mechanisms, including mitochondrial dysfunction, energy depletion, oxidative stress, and dysregulated inflammatory responses [6, 7]. Treatment strategies for SIC, as per guidelines, predominantly emphasize source control of infection, administration of broad-spectrum antibiotics, and supportive care. However, there is currently no specific intervention targeted at halting the progression of SIC. Therefore, identifying and advancing potential diagnostic biomarkers and treatment targets for SIC is crucial to reduce its mortality rate.

In recent years, dysfunction in endoplasmic reticulum stress (ERS) in the myocardial cells contributes to the progress and development of sepsis [8, 9]. ERS is characterized by the imbalanced accumulation of misfolded or unfolded proteins caused by cellular stress and inflammation [10]. It has been demonstrated that prolonged ERS facilitates inflammation, elevates the production of reactive oxygen species, promotes M1 macrophage polarization, and induces myocytes apoptosis [11]. In fact, the relationship between ERS and cardiomyopathy has been increasingly revealed. Liu et al indicated that circulating interleukin-1β promotes ERS-induced myocyte apoptosis in diabetic cardiomyopathy [12]. In addition, ERS was also demonstrated to promote iNOS/NO and influence inflammation in the development of doxorubicin-induced cardiomyopathy [13]. However, the possible relationship between ERS and SIC, especially the potential role of ERS in immune cell infiltration during SIC, remains unclear.

This study elucidated key ERS-related molecular mechanisms and their correlation with biologics in SIC pathogenesis. Additionally, we performed enrichment analysis, evaluated immune infiltration, and developed a machine learning model to illustrate the influence of ERS on SIC. The elucidation of the interplay between SIC and ERS holds promise for enhancing the comprehension of SIC pathomechanisms and providing clinicians with novel insights for therapeutic approaches.

## Materials and methods

### Data resource and differential analysis

The gene expression profile GSE79962 and corresponding clinical data were retrieved from the Gene Expression Omnibus (GEO) database (http://www.ncbi.nih.gov/geo). A total of 1,350 ERS-related genes (ERSRGs) with correlation scores greater than 5 were extracted from the GeneCards database (https://www.genecards.org/). Subsequently, differentially expressed genes (DEGs) were identified from the GSE79962 microarray data using the R package “limma” with a significance threshold of p < 0.05 and absolute log2 fold change (|log2 FC|) greater than 1. Heatmaps of the DEGs were generated using the R package “ggplot2”.

### Analysis of immune infiltrating patterns in SIC

We employed the CIBERSORT algorithm to assess the abundance of 22 immune cell types in the cardiac myocardial tissue of patients with SIC and healthy controls [14]. Differences between the two cohorts were determined using Student’s t-test, and the visual representation of these variances was generated through the “ggboxplot” R package. Furthermore, we employed the “corrplot” R package to examine the correlation between differentially expressed ERS-related genes (DE-ERSRGs) and immune cells, while the “pheatmap” R package was utilized to illustrate the findings.

### Weighted gene co-expression network analysis (WGCNA)

We performed WGCNA using the WGCNA package in R software, which builds scale-free co-expression networks for clinical phenotypes [15]. Firstly, discrete cases were filtered through hierarchical clustering analysis. Next, we selected an appropriate soft power β for subsequent weighted adjacency matrix construction, which was transformed into a topological overlap matrix containing module assignments that were labeled by module feature (ME) and color. Furthermore, we calculated the Pearson correlation coefficients to assess the correlation between ME and clinical characteristics. Finally, we demonstrated the most significant module related to the disease, and extracted the characteristic genes for subsequent analysis.

### Identification of ERS-related genes

Hub genes were identified by screening for genes from the hub module with the highest relevance to SIC in WGCNA. Venn diagrams of hub genes, DEGs, and ERGs were generated using the online tool “jvenn” (https://jvenn.toulouse.inrae.fr/app/example.html) to illustrate the overlapping genes, which were defined as DE-ERSRGs and further analyzed.

Subsequently, a protein-protein interaction (PPI) network was constructed using the STRING database version 11.5 (http://string-db.org/) to explore the relationships among DE-ERSRGs [16]. The PPI network data of DE-ERSRGs was then visualized using Cytoscape software version 3.8.2 (https://cytoscape.org) [17] with plugins MCODE and Cytohubba-MCC to identify significant gene clusters with scores equal to or greater than the median score of 8 [18]. Additionally, key transcription factors were identified using the iRegulon plugin [19]. Lastly, the protein-chemical interactions were analyzed utilizing data from the Comparative Toxicogenomics Database [20].

### The expression and correlation analysis of DE-ERSRGs

The expression profiles of DE-ERSRGs were visualized through a heatmap created using ComplexHeatmap. A comparison of the expression levels of DE-ERSRGs in cardiac samples from sepsis patients and normal controls, along with their chromosomal locations, was presented using the RCircos package in R. Furthermore, a receiver operating characteristic curve (ROC) prediction model was developed utilizing the pROC package to assess the diagnostic performance of DE-ERSRGs. The corrplot package was employed to analyze the correlations among DE-ERSRGs in order to elucidate their synergistic effects.

### Functional enrichment analysis

The clusterProfiler package was utilized to conduct Gene Set Enrichment Analysis (GSEA) using the “c2.cp.v7.2.symbols.gmt” gene set as the reference, with the ggplot2 package employed for result visualization. A significance level of P < 0.05 was considered statistically significant. Gene Set Variation Analysis (GSVA) [21] was employed to determine the enrichment scores of specific gene sets in each sample. Utilizing the c2.cp.v7.4.symbols.gmt dataset, the expression profiles of SIC patients were evaluated to analyze relevant biological characteristics, visualize changes in associated pathways through heatmaps and volcano plots.

Furthermore, the clusterProfiler package in R was applied for Gene Ontology (GO) and Kyoto Encyclopedia of Genes and Genomes (KEGG) pathway enrichment analyses in DE-ERSRGs, with a significance threshold of P < 0.05 [22]. The ggplot2 and GOplot packages were chosen for results visualization.

### Clinical diagnostic applications based on DE-ERSRGs

The least absolute shrinkage and selection operator (LASSO) technique was applied to select the optimal subset of DE-ERSRGs features, aiming to prevent the introduction of predictive optimism. Subsequently, a logistic regression model was developed to transform the selected DE-ERSRGs into a prediction score for SIC, and its effectiveness was confirmed through calibration curve analysis. Following the generation of the score, SIC patients were stratified into high- and low-risk groups based on the median score, and the expression levels of the remaining DE-ERSRGs were compared between these risk subgroups.

### Clinical prognosis analysis

The GSE65682 dataset was selected for survival analysis. Initially, based on the mean expression value of DE-ERSRGs, the samples in the GSE65682 dataset were stratified into high and low expression groups. DE-ERSRGs with significant implications for survival outcomes were further investigated, leading to the selection of the PDHB gene for subsequent research. Subsequently, Cox regression analysis for PDHB on the clinical factors of GSE65682 was carried out using the survminer and survival packages in R. The results were visualized using a forest plot to examine the relationship between clinical parameters and the prognostic value of PDHB. Finally, a nomogram plot was developed based on the multivariate Cox model to predict the 4-week survival of septic patients, and the accuracy of the nomogram was assessed through calibration curve analysis.

### Animal treatment

Male C57BL/6J mice aged 6–8 weeks and weighing 20–25 g were used in this study. The mice were housed under specific pathogen-free conditions with a 12-hour light/dark cycle, at a temperature of 23 ± 2°C and a relative humidity of 50 ± 10%. They had ad libitum access to food and water. The study was approved by the Institutional Animal Ethics Committee of the Second Affiliated Hospital of Fujian Medical University (Approval number: 2023698; Date: December 2023). Acute polymicrobial peritonitis was induced in the mice to create the SAE model through cecum ligation puncture (CLP). Anesthesia was administered to all mice with a preoperative intraperitoneal injection of 1% sodium pentobarbital (40 mg/kg, Sigma-Aldrich, St Louis, MO, USA). The cecum was ligated and double-punctured below the ileocecal region using a 22-gauge needle. Mice in the sham-operated group underwent a similar procedure without CLP induction. Throughout the surgery, the mice’s body temperature was maintained at 37 ± 0.5°C until they recovered, after which they were returned to their cages. On the 7th day post-operation, mice were euthanized by neck dissection to collect heart samples.

### Real-time quantitative real-time polymerase chain reaction analysis (qPCR)

According to the results of the aforementioned analysis, the mRNA expression of PDHB was validated by qPCR in CLP-induced systemic inflammatory response syndrome (SIRS) mice. Briefly, RNA was extracted from cardiac tissues using TRIzol (GeneCopoeia, MD, USA), followed by reverse transcription of 1 μg total RNA from each sample into complementary DNA (cDNA) using specific primers and SYBR Green reaction mix (Takara Biotech). Real-time qPCR was conducted on the Bio-Rad Real-Time PCR cycler, and gene expression was quantitatively analyzed using the 2^^-ΔΔCt^ method. The primer sequences used were: PDHB forward: AATCATCTCGTGAC TGTGGAAGGAG, reverse: GGCATAGG-GACATCAGCACCAG; GAPDH forward: GGCAAATTCAACGGCACAGTCAAG, reverse: TCGCTCCTGGAAGATGGTGA-TGG.

### Western blotting

In short, proteins were separated using 10% SDS-PAGE after being quantified with the BCA reagent, followed by their transfer to a PVDF membrane (Millipore, Billerica, MA, USA). Subsequently, the membranes were blocked with 5% skimmed milk at room temperature for 2 hours, incubated overnight with anti-β-actin (Affinity, AF7018, 1:10000) and anti-PDHB (Abways, CY7208, 1:2000) primary antibodies at 4°C. After that, the membranes were treated with a horseradish peroxidase-conjugated secondary antibody at room temperature for 1 hour, and the ChemiDoc imaging system (Bio-Rad) was utilized to capture the images.

### Statistical analysis

Statistical analyses were conducted using R Studio version 4.0.2. The student’s t-test assessed changes in normally distributed continuous variables between groups, while the Mann-Whitney U test (Wilcoxon rank-sum test) was used for variables with non-normal distribution. All tests were two-sided, and significance was set at P < 0.05.

## Results

### Weighted gene co-expression network analysis

**Fig 1:**
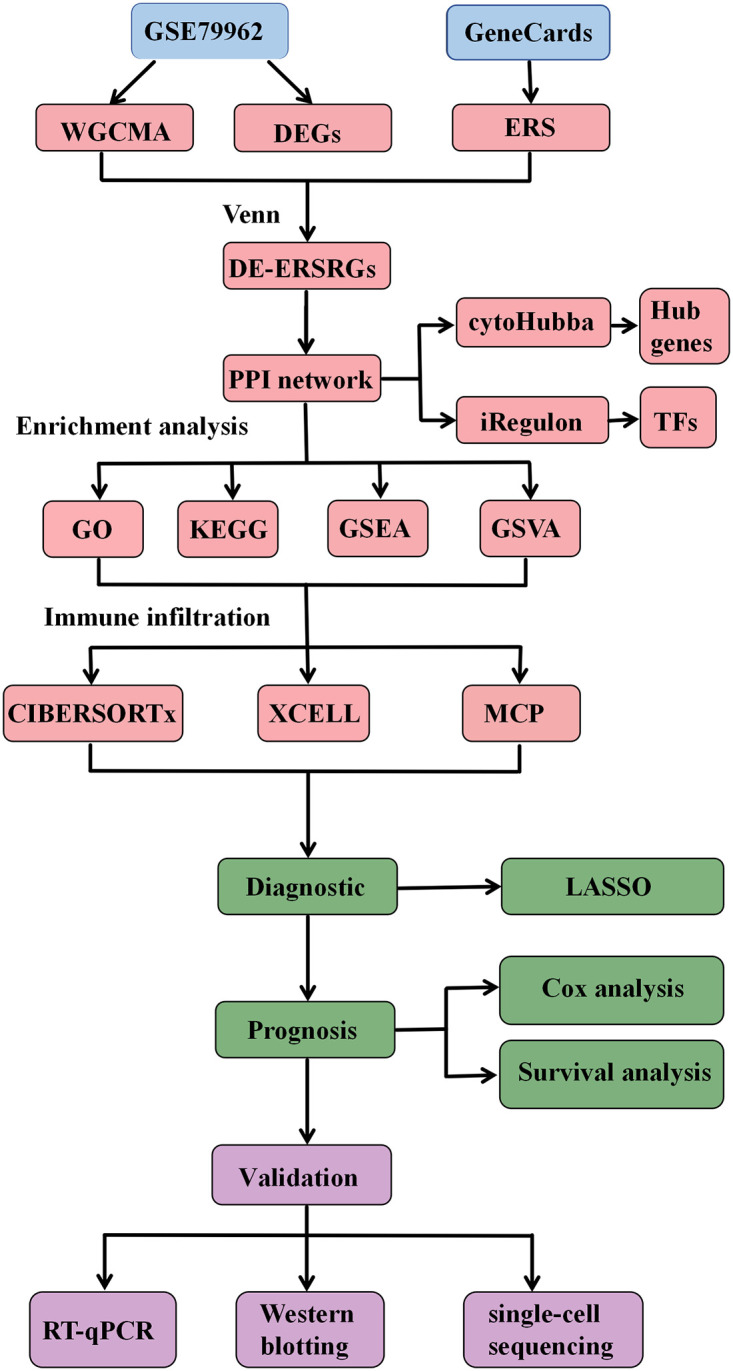
Flow chart of the study process.

**Fig 2:**
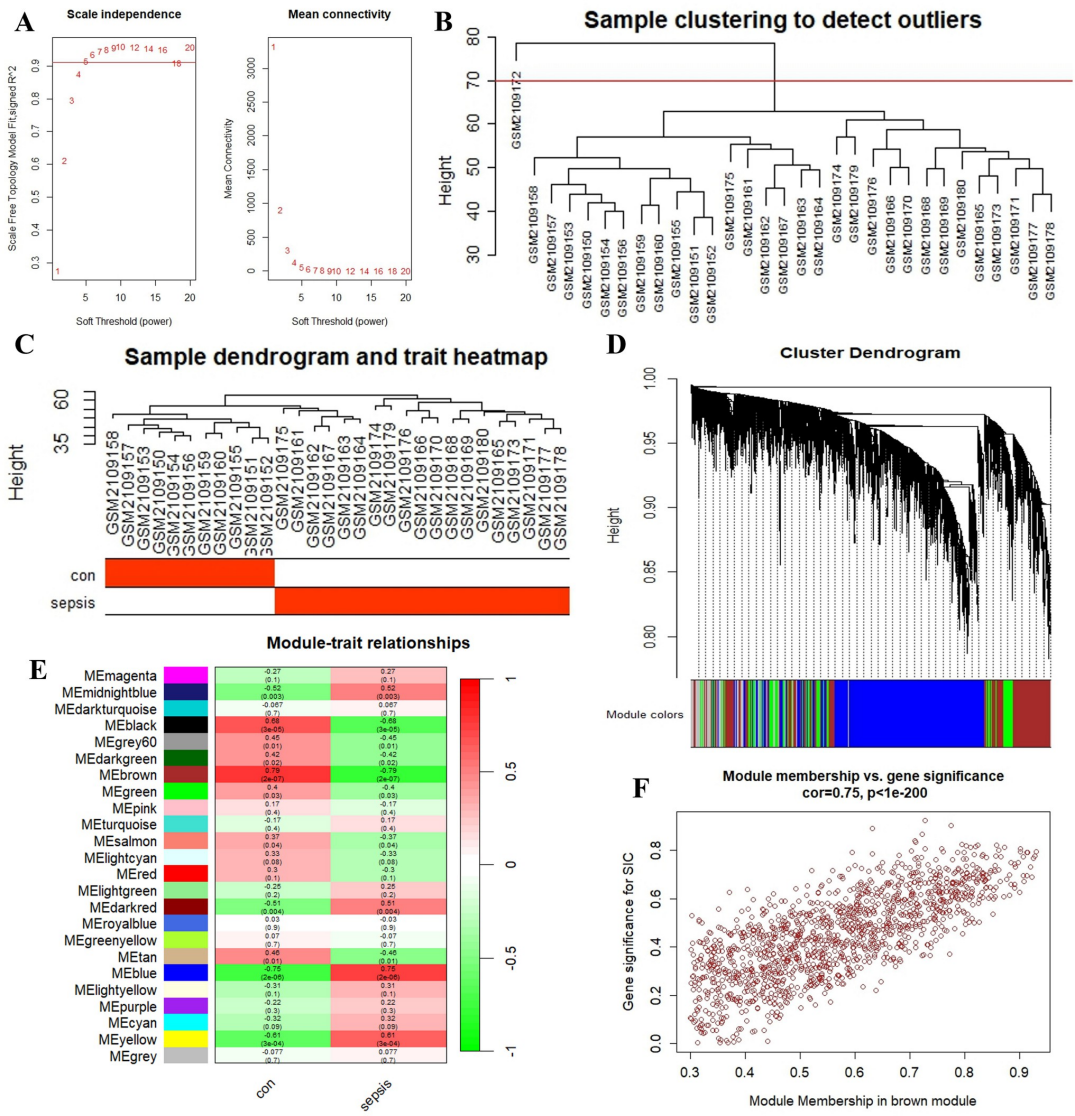
WGCNA selection of SIC disease-related modules co-expressed genes. (A) Network topology analysis under various soft-threshold powers; (B) Outliers were detected in the sample cluster; (C) Sample clustering dendrogram based on Euclidean Distance; (D) Clustering dendrogram of genes with different similarities based on topological overlap and the assigned module color; (E) Heat map of the correlation between module characteristic genes and phenotypes; (F) The relevance of members in the brown module and SIC.

The flow chart is shown in Fig 1. To explore the co-expression relationships between genes and phenotypes, we utilized the WGCNA package in R to construct a gene co-expression network. Following the cluster analysis, the outlier sample GSM2109172 was excluded (Fig 2B and 2C). A key soft threshold of β = 5 was chosen to ensure the scale-free nature of the co-expression module (Fig 2A). A total of 24 gene co-expression modules were identified in the module-trait relationship between the sepsis and control groups (Fig 2E), represented by color-coded gene clusters in Fig 2D. Notably, the brown module showed the highest correlation (cor = 0.79, p = 2e-07) and was identified as a hub module. The scatter plot of the brown module illustrated the relationship between module membership and gene significance, with a correlation coefficient (cor) of 0.75 (p<1e-200) (Fig 2F). This observation suggests that as the number of genes in the brown module increases, the correlation between the gene and SIC, as well as its importance in the studied trait, also rises.

### Identification of key DE-ERSRGs in sepsis-induced cardiomyopathy

**Fig 3:**
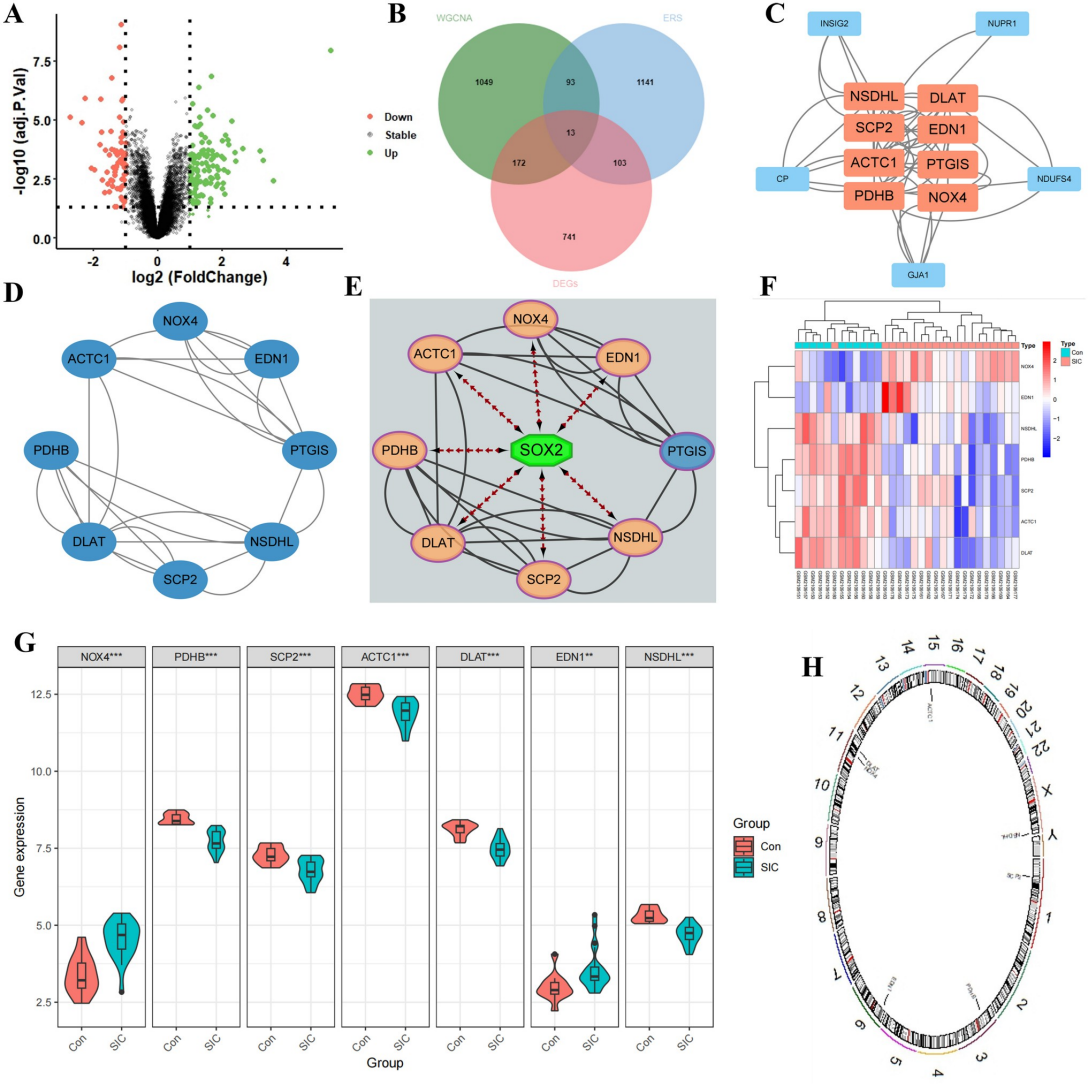
Differential analysis of SIC. (A) Volcano graph of the control group and SIC group in differential analysis; (B) Venn figure for intersected genes in DEGs, WGCNA and ERS; (C) PPI network diagram of hub genes; (D) PPI network diagram of hub genes under Degree algorithm; (E) Hub genes-TF interaction network; (F) Heat map of DE-ERSRGs in SIC and control groups; (G) Comparison of DE-ERSRGs in SIC and control groups; (H) Chromosome location map of DE-ERSRGs.

**Fig 4:**
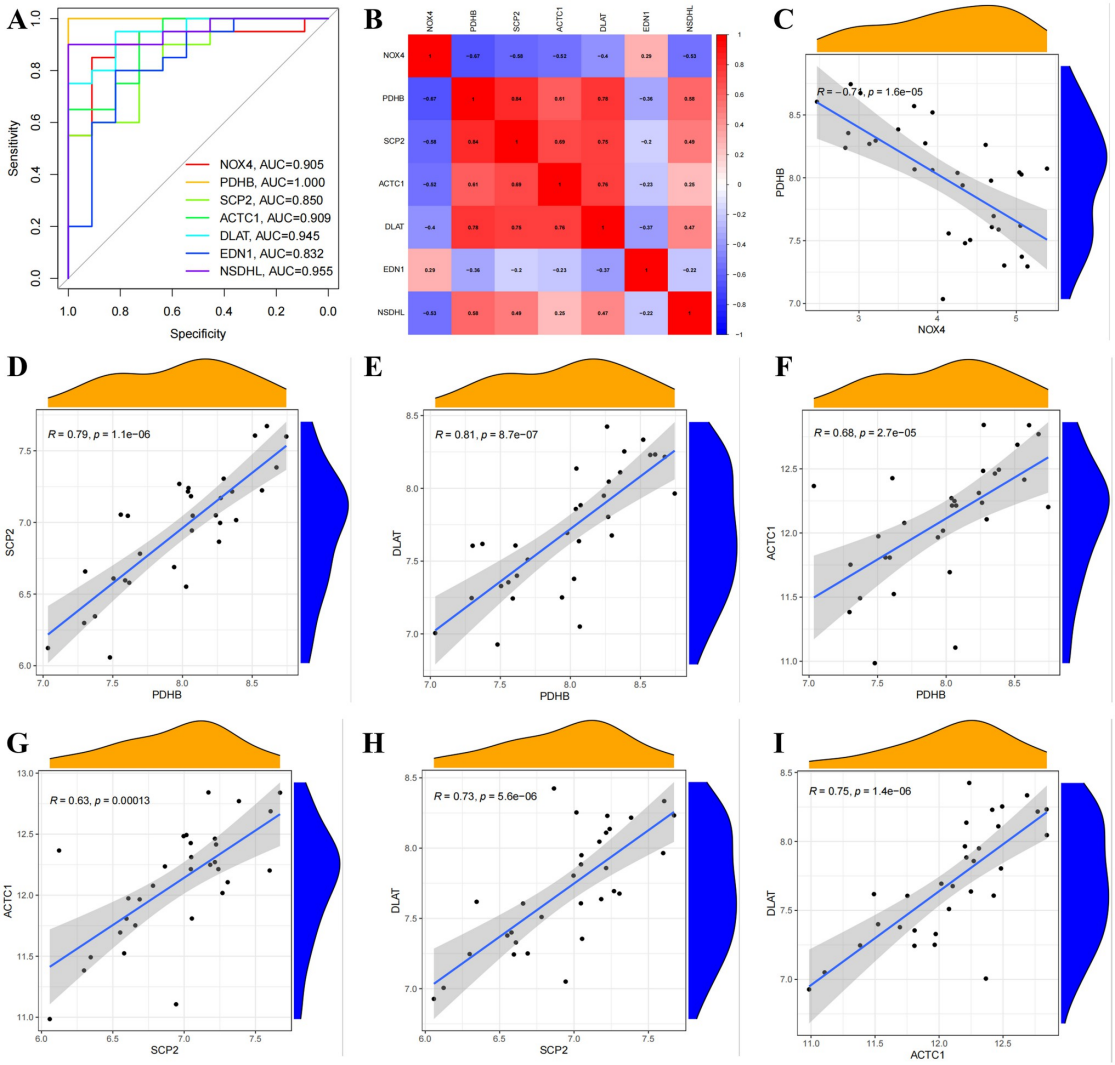
Correlation analysis of DE-ERSRGs. (A) ROC analysis of DE-ERSRGs; (B) Heat map of the correlations among DE-ERSRGs; (C) The correlations between PDHB and NOX4; (D) The correlations between PDHB and SCP2; (E) The correlations between PDHB and DLAT; (F) The correlations between PDHB and ACTC1; (G) The correlations between SCP2 and ACTC1; (H) The correlations between SCP2 and DLAT; (I) The correlations between DLAT and ACTC1.

The selection criteria utilized were |log2FC| > 0.5 and p-value < 0.05, resulting in 1031 DEGs, with 544 genes showing high expression and 487 with low expression(Fig 3A). The intersection of these 1031 DEGs with GSE79962 (S1 Table), 1327 brown modular genes (S2 Table), and 1350 ERSRGs identified 13 DE-ERSRGs (Fig 3B, S3 Table). Subsequently, a PPI network was constructed using the STRING database, revealing 8 genes with a degree > = the median score of 8 identified through the cytoHubba-MCC plugin (Fig 3C and 3D, S4 Table). The iRegulon plugin in Cytoscape software was employed to explore the transcription factor (TF) binding patterns of the 8 genes, showing that seven genes are regulated by SOX2, leading to their selection for further analysis (Fig 3E). Fig 3F and 3G depicted the expression patterns of the 7 DE-ERSRGs between the healthy controls and SIC group, with their chromosomal loci presented in Fig 3H. Furthermore, ROC analysis was conducted to illustrate the diagnostic significance of these DE-ERSRGs (Fig 4A), indicating that all genes had an AUC > 0.800, with EDN1 exhibiting the smallest AUC value (AUC, 0.832) and PDHB the largest (AUC, 1.000). Additionally, correlations among ERSRGs were explored via a correlation heat map (Fig 4B), and scatter plots were used to display the interactions among genes with significant synergistic effects (Fig 4C–4I).

### Functional classification and pathway enrichment of DEGs

**Fig 5:**
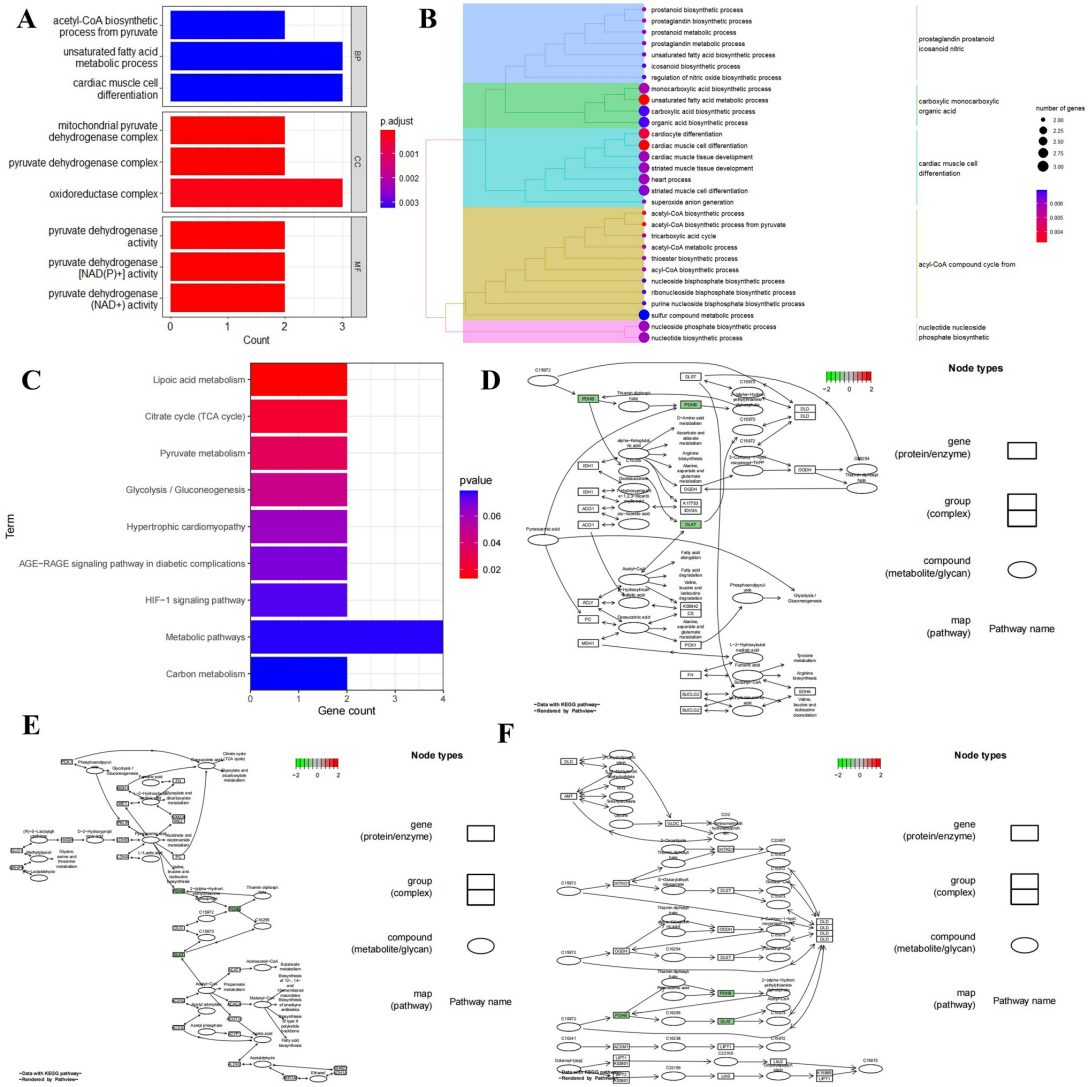
GO and KEGG analysis. (A) GO functional enrichment analysis of hub genes; (B) Bubble plot of GO enrichment analysis; (C) KEGG pathway enrichment analysis of DE-ERSRGs; (D) Pathway map of Lipoic acid metabolism; (E) Pathway map of Citrate cycle (TCA cycle); (F) Pathway map of Pyruvate metabolism.

To delve deeper into the functional effects of the 7 DE-ERSRGs, we initially conducted GO and KEGG analyses (S5 and S6 Tables). The significant functional effects identified in the top-ranked groups included acetyl-CoA biosynthetic process from pyruvate, unsaturated fatty acid metabolic process, mitochondrial pyruvate dehydrogenase complex, pyruvate dehydrogenase activity, pyruvate dehydrogenase [NAD(P)+] activity, lipoic acid metabolism, and Citrate cycle (TCA cycle) (Fig 5A–5C). Notably, the results of the functional enrichment analyses suggested a strong association of the 7 DE-ERSRGs with glucose metabolism. Subsequent Pathview package analysis based on the KEGG data revealed significant expressions of DE-ERSRGs in the Lipoic acid metabolism (Fig 5D), Citrate cycle (TCA cycle) (Fig 5E), and Pyruvate metabolism (Fig 5F) signaling pathways.

### Gene set enrichment analysis and gene set variation analysis

**Fig 6:**
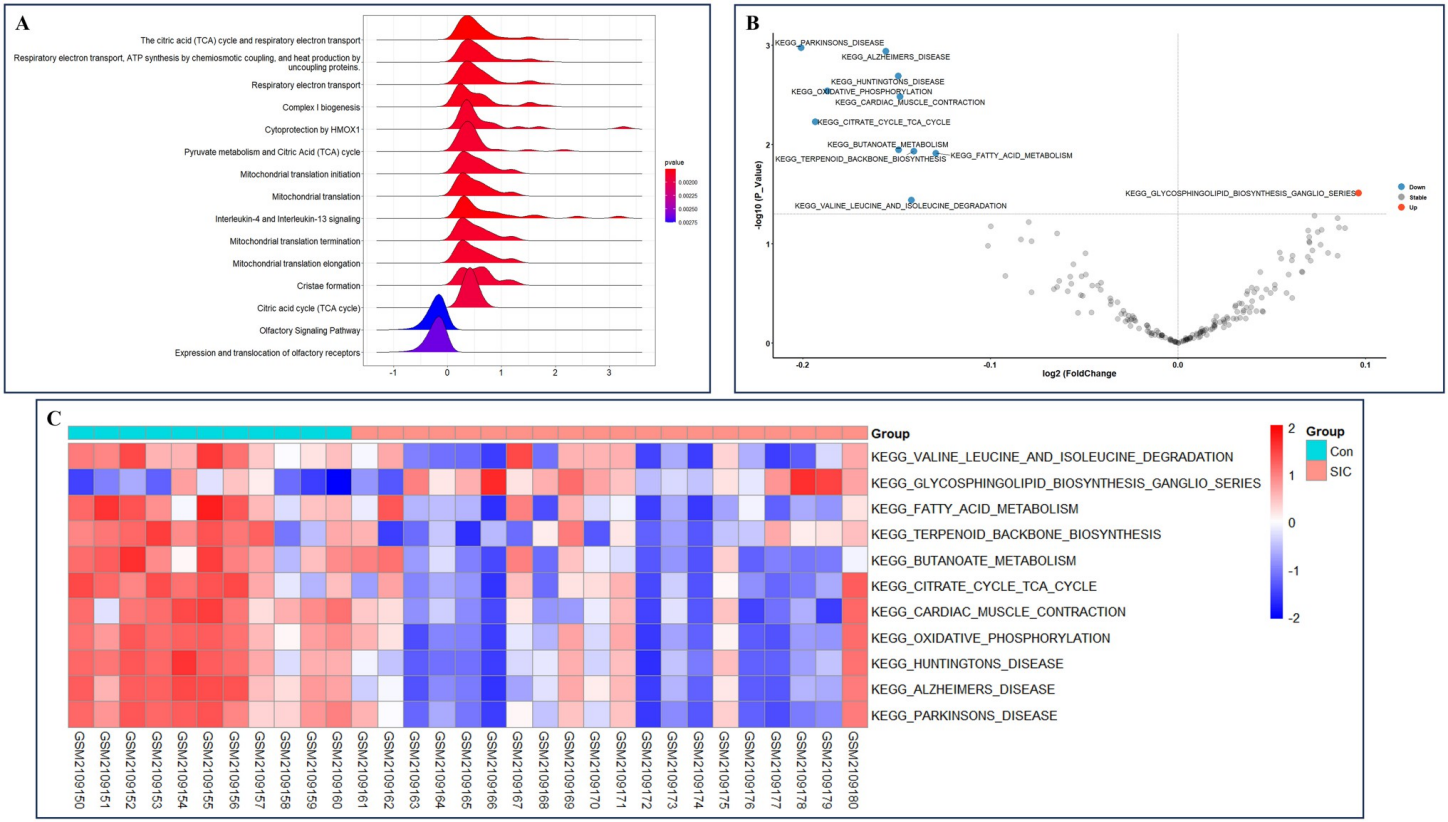
GSEA and GSVA enrichment analysis. (A) GSEA enrichment analysis of the main 15 biological characteristics; (B) Volcano graph of GSVA; (C)Heat map of GSVA.

In order to overcome the limitations arising from relying solely on the intersection gene enrichment, we conducted GSEA by incorporating all genes from GSE79962 (S7 Table). The analysis revealed significant enrichment of genes in pathways related to glucose metabolism, such as the citric acid (TCA) cycle and respiratory electron transport (Fig 6A). Subsequently, to investigate the pathways with significant differences between the samples from the SIC and control groups in the combined dataset, we utilized GSVA to assess the functional enrichment disparities of each sample between the SIC and control groups (S8 Table). The GSVA results highlighted significant variations in pathways, including valine, leucine, and isoleucine degradation, glycosphingolipid biosynthesis ganglion series, fatty acid metabolism, TCA cycle, cardiac muscle contraction, oxidative phosphorylation, and others between the SIC and control groups (Fig 6B). Notably, only the pathway of glycosphingolipid biosynthesis ganglion series showed upregulation in the SIC group, while the remaining pathways exhibited downregulation (Fig 6C).

### Construction of chemical-hub gene networks for SIC

**Fig 7:**
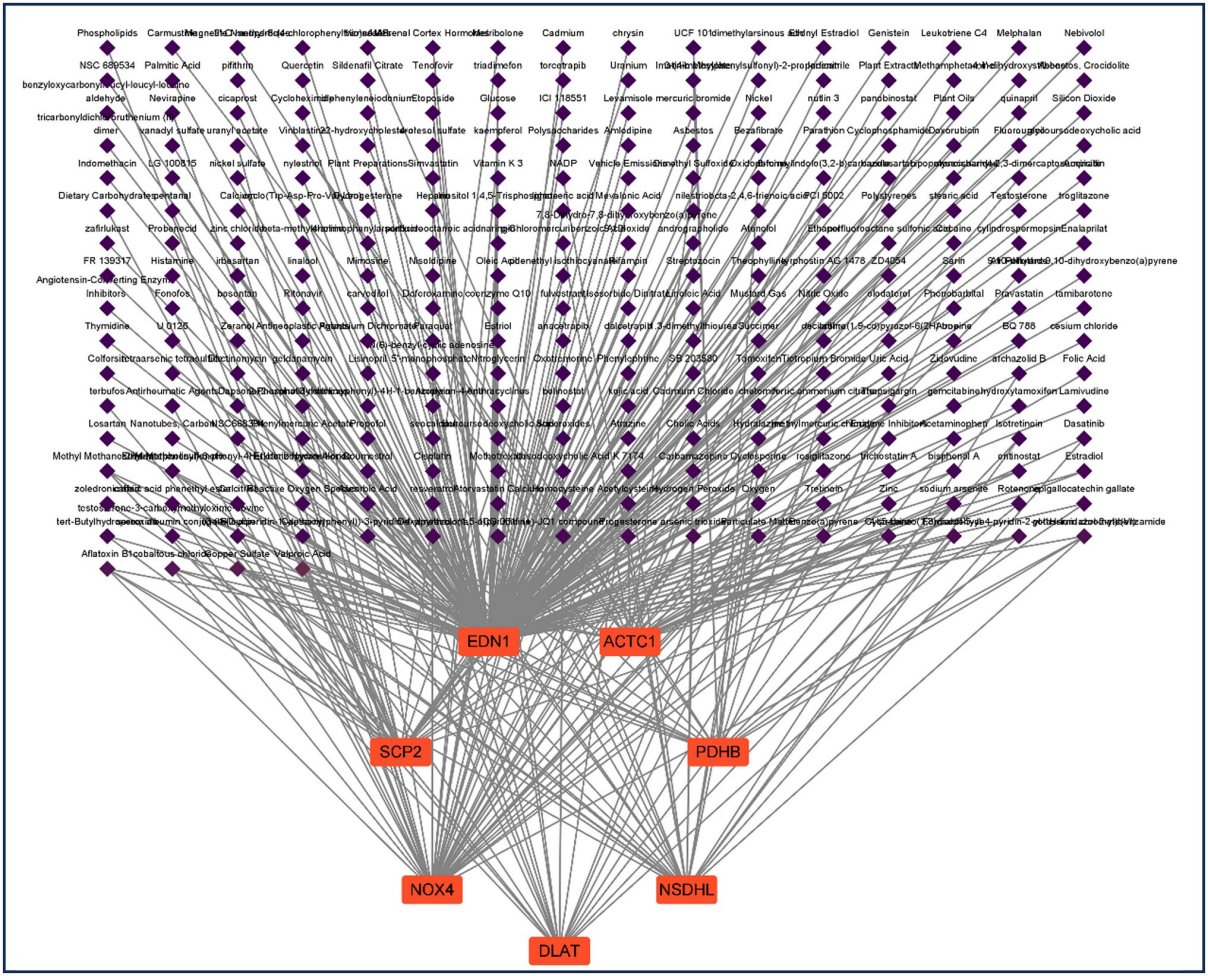
DE-ERSRGs-chemical interactive network.

We then constructed the interaction network between the 7 DE-ERSRGs and chemicals using the Comparative Toxicogenomics Database, identifying 351 chemicals (Fig 7).

### Immune cell infiltration in sepsis-induced cardiomyopathy

**Fig 8:**
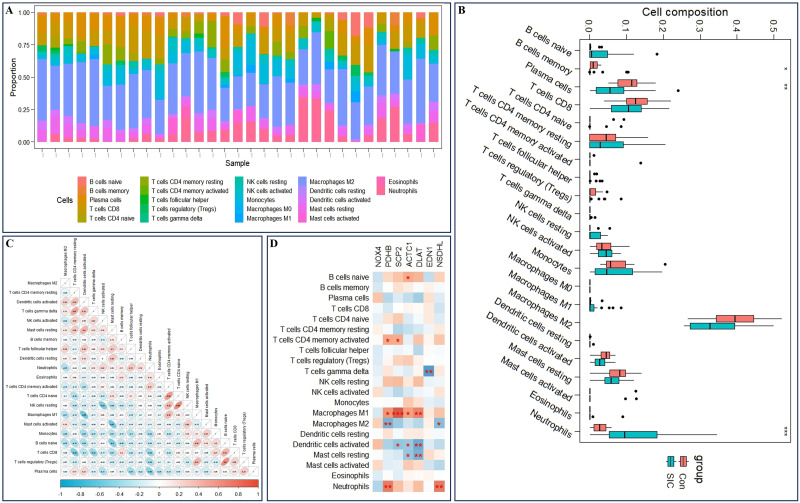
Immuno-infiltration analysis. (A) A histogram of immune cell infiltration; (B) Distribution box diagram of immune cells in high and low risk groups; (C) Correlation heat map of immune cell infiltration abundance; (D) Correlation heat map between ERSRGs and immune cell.

The CIBERSORTx algorithm was utilized to determine the correlation between 22 immune cells and the expression profiles of the SIC and control groups in the GSE79962 dataset. Initially, the immune infiltration results for each sample in both groups were graphically displayed using histograms (Fig 8A, S9 Table). To delve deeper into the relationship between SIC and immune cells, we quantified the scores of 22 immune cells in the SIC group using the CIBERSORTx algorithm (Fig 8B). Neutrophils exhibited the most significant increase in the SIC group compared to the Con group (P < 0.001). Subsequently, we validated the observed increase in neutrophil levels in SIC using two distinct algorithms (XCELL and MCP) and confirmed the rise in neutrophils (S2 Fig, S10 and S11 Tables; P < 0.05). Further analysis involved examining the correlation among the infiltration levels of these immune cells (Fig 8C), prompting the removal of one immune cell (Macrophages M0) that displayed no significant correlation with other immune cells. The remaining 21 immune cells were then assessed for correlation with the 7 previously obtained DE-ERSRGs, revealing a notable correlation between 17 gene pairs and immune cells as depicted in the heatmap (Fig 8D).

### Clinical diagnostic applications based on ERSRGs

**Fig 9:**
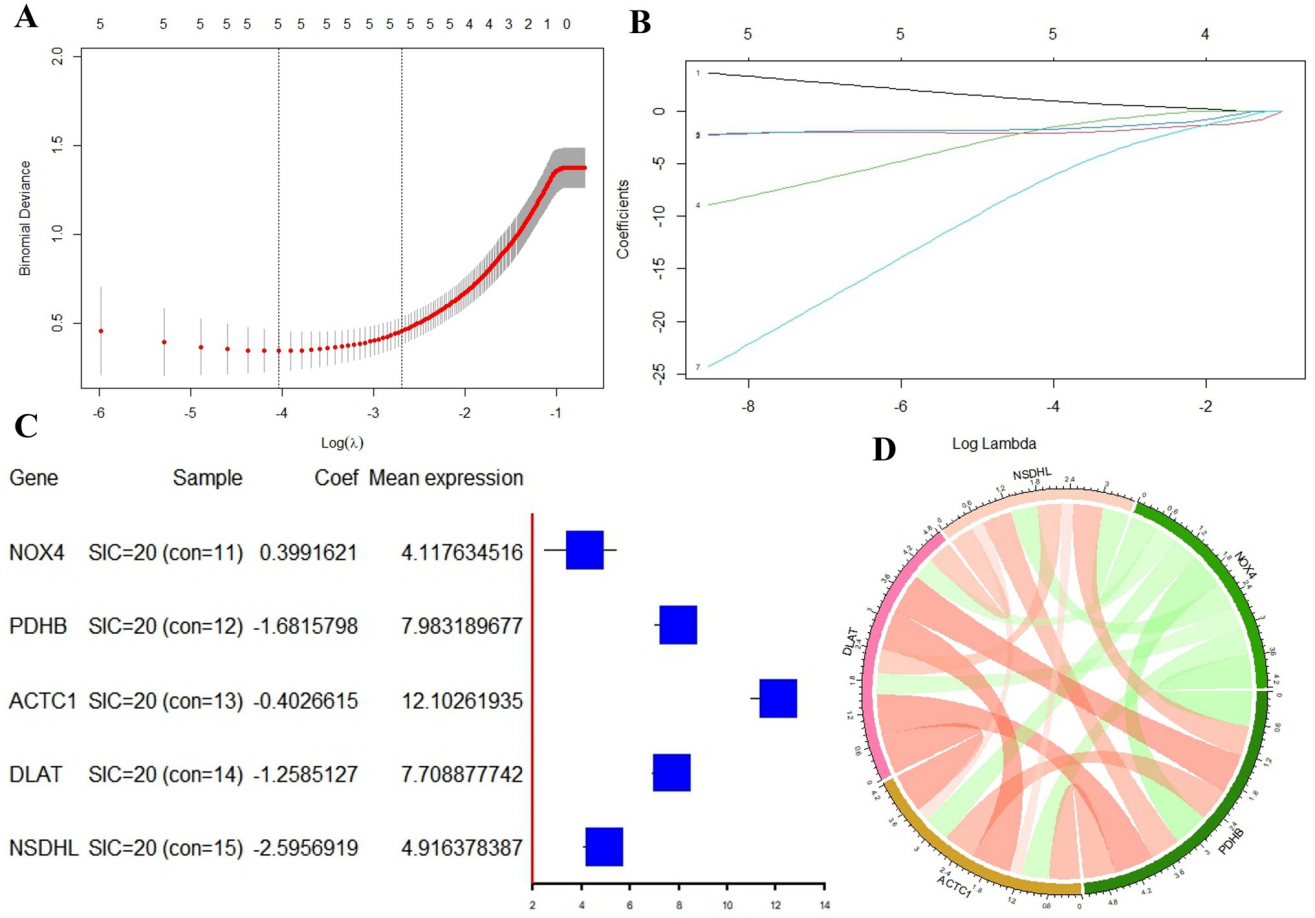
Construction of diagnostic model of ERSRGs. (A) LASSO regression diagnostic model diagram; (B) LASSO variable locus diagram; (C) Forest map of ERSRGs; (D) Gene expression correlation chord diagram.

We utilized LASSO regression analysis to develop a diagnostic model for DE-ERSRGs in order to assess their diagnostic significance. Five DE-ERSRGs (NOX4, PDHB, ACTC1, DLAT, NSDHL) were identified as having diagnostic value (refer to Fig 9A). A LASSO variable trajectory was plotted to visually represent the results of the LASSO regression (see Fig 9B). Subsequently, using a forest plot (Fig 9C), we illustrated the expression levels of the five genes identified by LASSO across different groups (SIC and control). Notably, in Fig 9C, ACTC1 displayed the highest average expression level. Additionally, a correlation circle diagram was utilized to demonstrate the expression correlations among the genes. In Fig 9D, the diagram reveals that the positive correlation between PDHB and DLAT is the strongest, while the negative correlation between PDHB and NOX4 is the most prominent.

**Fig 10:**
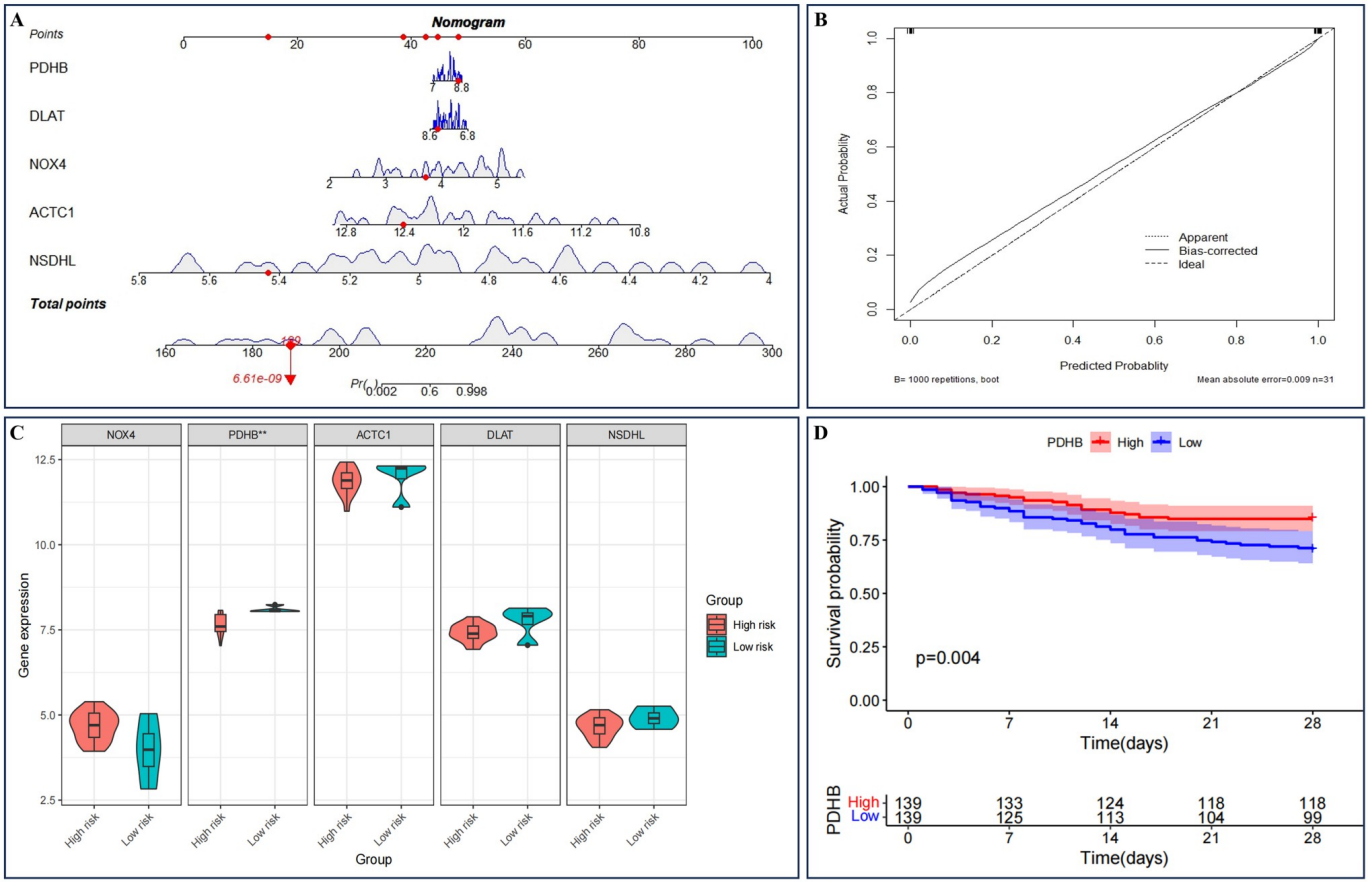
Cox model of LASSO model gene. (A) nomogram of single-factor and multifactor Cox regression analysis of LASSO diagnostic model; (B) Calibration curve; (C) LASSO model gene expression difference analysis in different risk groups comparison box diagram; (D) The survival curve of PDHB.

Following this, a logistic regression model was established to transform the five DE-ERSRGs identified through LASSO regression into SIC prediction scores, which were then used to create a nomogram (Fig 10A). The model’s effectiveness was assessed through a calibration curve (Fig 10B). Subsequently, based on the average scores, SIC patients were categorized into high-risk and low-risk groups, and a comparison of the expression levels of the five DE-ERSRGs between the two groups was conducted. Results indicated that PDHB exhibited the most significant difference between the different risk groups (see Fig 10C).

### Clinical prognosis analysis of the DE-ERSRGs

We chose the new GEO dataset GSE65682 to conduct survival analysis on the five previously obtained DE-ERSRGs. Among them, only PDHB exhibited a significant correlation with the survival of sepsis patients (refer to Fig 10D and S1 Fig); hence, PDHB was singled out for further investigation. Subsequently, utilizing the clinical data from GSE65682 (refer to Table 1), we delved into analyzing the impact of other clinical factors on survival likelihood.

**Table 1:** Basic clinical characteristics in patients with high or low PDHB.

	Overall	High	Low	P value
n	278	133	145	
Gender (%)				1.0
female	129 (46.4)	62 (46.6)	67 (46.2)	
male	149 (53.6)	71 (53.4)	78 (53.8)	
Age		59.26 (15.81)	63.27 (13.14)	0.022
<63 yr	132 (47.5)	69 (51.9)	63 (43.4)	0.198
>=63 yr	146 (52.5)	64 (48.1)	82 (56.6)	
Endotype class (%)				<0.001
Mars1	78 (28.1)	16 (12.0)	62 (42.8)	
Mars2	101 (36.3)	64 (48.1)	37 (25.5)	
Mars3	68 (24.5)	43 (32.3)	25 (17.2)	
Mars4	31 (11.2)	10 (7.5)	21 (14.5)	
ICU acquired infection (%)				0.969
ICUA	41 (14.7)	19 (14.3)	22 (15.2)	
No ICUA	237 (85.3)	114 (85.7)	123 (84.8)	
Diabetes mellitus (%)				0.23
DM	62 (22.3)	25 (18.8)	37 (25.5)	
No DM	216 (77.7)	108 (81.2)	108 (74.5)	
PDHB (mean ± SD)	6.20 ± 0.53	6.63 ± 0.26	5.79 ± 0.37	<0.001

PDHB, Pyruvate dehydrogenase B; DM, Diabetes mellitus; ICUA, intensive care unit acquired infection; SD, standard deviation.

**Fig 11:**
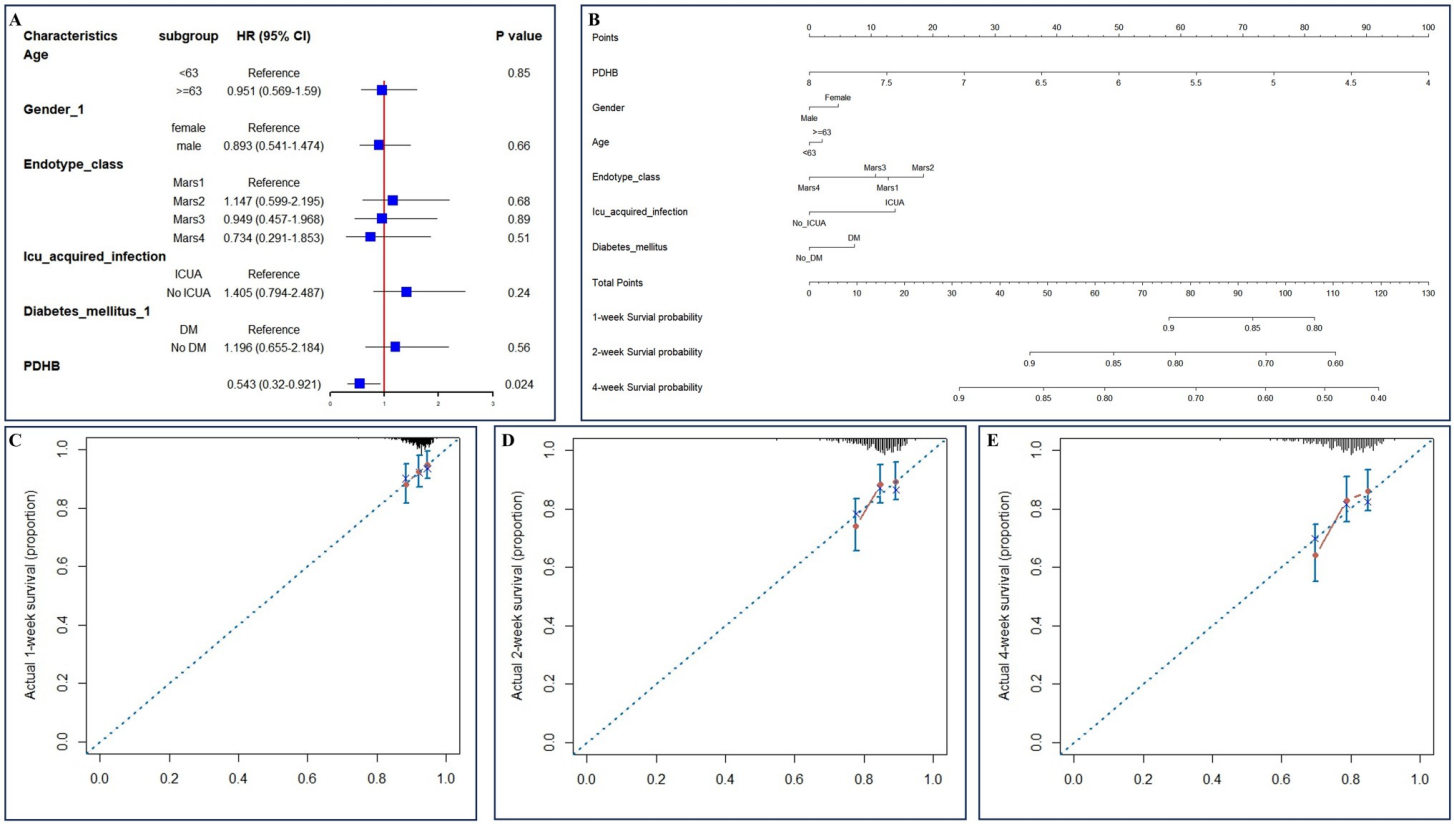
Clinical prognosis analysis. (A) Forest plot for multivariate Cox regression analysis; (B) Nomogram plot for multivariate Cox regression analysis; (C-E) 1 week, 2 week and 4week survival rate calibration curve.

PDHB underwent univariate and multivariate Cox regression analyses, encompassing variables such as age, gender, different endotype classes, whether the infection was acquired in the intensive care unit (ICU), and the presence of diabetes (refer to Table 2). The outcomes of the multivariate Cox analysis revealed a significant correlation between PDHB and survival rates (see Fig 11A). To evaluate the predictability of clinical factors on survival rates, particularly one-week, two-week, and four-week survival rates, a nomogram was established based on the multivariate findings (see Fig 11B). The calibration curves indicated that the nomogram’s performance was satisfactory (Fig 11C–11E).

**Table 2:** Univariate and multivariate Cox analysis.

Variables	Univariate analysis		Multivariate analysis	
	Log rank X^2^	*P*	HR (95% CI)	P value
Age (years)	0	0.9	0.9629 (0.5719–1.6212)	0.8870
Gender	0	0.9	0.9048 (0.5465–1.4978)	0.6972
PDHB	993	**<0.001**	0.5834 (0.3697–0.9205)	**0.0206**
Endotype class	1.2	0.7	0.9186 (0.7128–1.1839)	0.5120
ICU acquired infection	1.6	0.2	1.4030 (0.7544–2.6095)	0.2848
Diabetes mellitus	0.2	0.6	1.1751 (0.6407–2.1551)	0.6021

Note: Univariate analysis, Kaplan–Meier analysis; multivariate analysis, cox regression analysis; HR: hazard ratio; ICU, intensive care unit.

### Validation of PDHB expression in SIC

**Fig 12:**
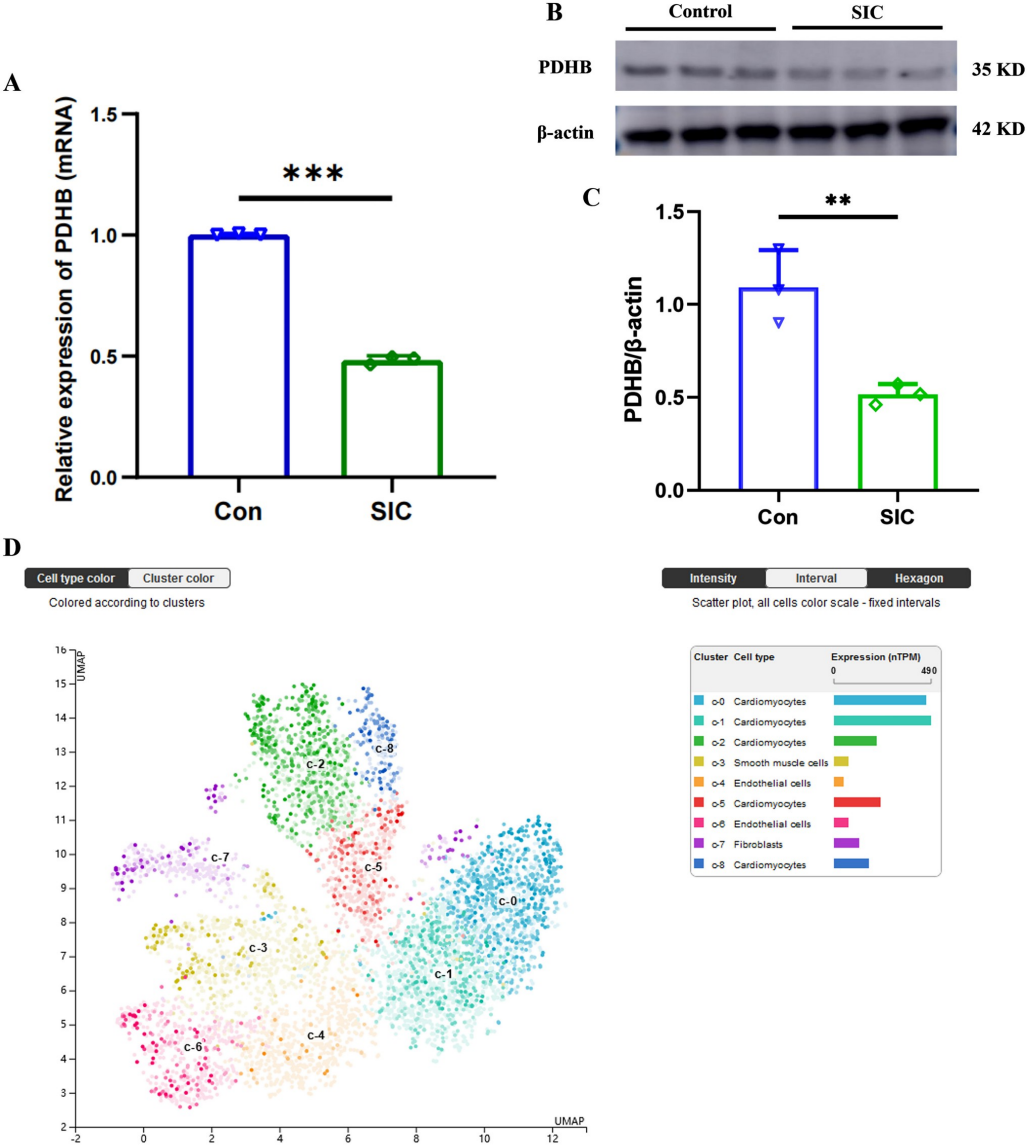
Experimental validation. (A) PDHB mRNA levels in heart tissue from SIC and control mice; (B-C) PDHB protein levels in heart from SIC and control mice; (D) Single-cell sequencing database results showing that PDHB is majorly expressed in the cardiomyocytes.

Based on the bioinformatics analysis, PDHB emerges as a crucial regulatory and prognostic factor linked to glucometabolic disorders and immune responses in SIC. To validate these observations, we induced a SIC model via CLP in C57BL6 mice and assessed the expression of PDHB within their heart tissues. The findings revealed that both mRNA and protein levels of PDHB were markedly lower in the SIC group compared to the control group (refer to Fig 12A–12C, S3 Fig). Additionally, the results from single-cell sequencing data retrieved from the Protein Atlas database highlighted predominant expression of PDHB in myocardial cells (see Fig 12D).

## Discussion

Sepsis is a systemic infectious disease characterized by multiple organ dysfunctions, resulting in poor survival rates and prognosis, making it a leading cause of hospital mortality [23]. SIC significantly elevates the mortality associated with sepsis [24], with numerous studies highlighting that mitigating cardiac damage can enhance treatment outcomes and lower mortality rates in sepsis patients [25–27]. The etiology of SIC remains elusive, and effective treatment modalities are lacking. The endoplasmic reticulum (ER), a crucial organelle involved in processing aberrant proteins intracellularly, is susceptible to endoplasmic reticulum stress (ERS) within an inflammatory milieu, triggering cardiomyocyte apoptosis [28]. Several reports have underscored the pathological role of ERS in sepsis, identifying the ERS-induced apoptosis pathway as a promising therapeutic target [29–32]. Nonetheless, the link between ERS and SIC warrants elucidation. Hence, our study aims to scrutinize the regulatory influence of ERS and the immune microenvironment on the onset and progression of SIC while identifying potential targets. These findings could aid in the identification of clinical biomarkers and therapeutic targets for SIC, shedding light on the molecular mechanisms underpinning SIC pathogenesis.

In this investigation, we identified 1031 Differentially Expressed Genes (DEGs) from the dataset GSE79962. Comparative analysis revealed that among these genes, 544 exhibited upregulation, while 487 showed downregulation in the samples with SIC compared to the control group. Subsequently, utilizing WGCNA, we identified 1327 genes highly associated with SIC and overlapped them with the DEGs and ERSRGs, leading to the discovery of 13 DE-ERSRGs. Further analyses involving PPI and TF binding pattern assessments resulted in the selection of seven DE-ERSRGs (NOX4, PDHB, SCP2, ACTC1, DLAT, EDN1, NSDHL) with robust correlations.

One of the primary pathological mechanisms underlying sepsis is the dysregulated immune response. Studies have shown that circulating innate immune cells, including monocytes, macrophages, neutrophils, and dendritic cells, can modulate the functions of tissue-resident immune cells, potentially leading to organ dysfunction through the initiation of systemic inflammatory responses [33, 34]. Moreover, diminished levels of adaptive immune cells like CD8+ and CD4+ T cells, as well as B cells, have been linked to the progression of sepsis [35]. Notably, our investigation revealed a heightened presence of neutrophils in the SIC samples compared to controls, suggesting their significant role in shaping the immune microenvironment associated with SIC. To delve deeper into the relationship between ERSRGs and immune responses in SIC, we assessed the correlation between immune cells and seven DE-ERSRGs. Analysis via correlation heat mapping revealed a strong association of six DE-ERSRGs with various immune cell types, indicating a potential pivotal role of ERSRGs in SIC development through immune cell modulation.

Furthermore, we noted a significant correlation between ERSRGs and metabolic processes in SIC based on the outcomes of enrichment analysis. During the initial stages of sepsis, the Warburg effect, characterized by a metabolic shift from oxidative phosphorylation to aerobic glycolysis, furnishes activated leukocytes with a rapid ATP source by converting pyruvate to lactate [36]. Simultaneously, metabolic byproducts from aerobic glycolysis can fuel cytokine production, antimicrobial responses, and cell proliferation. However, in the later phase, transitioning from aerobic glycolysis to fatty acid oxidation and a catabolic state can suppress the inflammatory response [37]. Therefore, the modulation of metabolic pathways plays a critical role in sepsis progression. In our study, the functional profiling of seven DE-ERSRGs revealed substantial enrichment in biological processes (such as acetyl-CoA biosynthetic process from pyruvate and unsaturated fatty acid metabolic process), molecular functions (like mitochondrial pyruvate dehydrogenase complex and pyruvate dehydrogenase complex), cellular components (comprising pyruvate dehydrogenase complex and pyruvate dehydrogenase activity), and biological pathways (including Lipoic acid metabolism and Citrate cycle (TCA cycle)), emphasizing the evident connection between DE-ERSRGs and metabolic alterations in SIC development.

This study assessed the diagnostic and prognostic relevance of seven DE-ERSRGs. Analysis of the ROC revealed that all DE-ERSRGs exhibited satisfactory AUC values exceeding 0.800, with EDN1 displaying the smallest AUC value (AUC, 0.832) and PDHB the highest (AUC, 1.000). Notably, only PDHB demonstrated remarkable prognostic utility in sepsis. Pyruvate dehydrogenase (PDH) serves as a pivotal rate-limiting enzyme in the tricarboxylic acid cycle, facilitating the conversion of pyruvate to acetyl-CoA and carbon dioxide [38]. Previous literature has documented that sepsis can diminish both the quantity and activity of PDH, culminating in elevated lactate levels [39, 40]. Conversely, activation of the PDH complex has been associated with maintaining immunometabolism homeostasis and enhancing sepsis survival [41]. PDHB, a crucial regulatory subunit of PDH [42], is yet to unveil its precise role in sepsis/SIC. Our investigation revealed its significant association with various immune cell types (including CD4 memory activated T cells, Macrophages M1/M2, and Neutrophils) through the CIBERSORTx algorithm, implying that PDHB may modulate immune-related endoplasmic reticulum stress by regulating glucose metabolism. We confirmed decreased PDHB mRNA and protein levels in the hearts of SIC model induced by CLP, with predominant expression in myocardial cells according to single-cell sequencing data from the Protein Atlas database. Further examination of PDHB expression patterns in LPS-stimulated H9C2 cardiomyocytes demonstrated a consistent profile to the SIC model using PCR and western blot analyses. Collectively, these findings underscore PDHB as a promising diagnostic/prognostic indicator for SIC, hinting at its crucial role in disease progression. Moreover, the regulatory function of PDHB in immune-related endoplasmic reticulum stress warrants further elucidation through functional and mechanistic experiments.

This study is not without limitations. Firstly, we solely examined PDHB expression through in vivo experiments without delving deeper into its associated pathway molecules. Secondly, additional animal studies are warranted to complement and validate the results obtained from other sequencing analyses, especially concerning immune cell infiltration. Lastly, validation in conjunction with clinical samples from SIC patients is essential.

## Conclusion

In conclusion, this study utilized integrated bioinformatics and machine learning algorithms to uncover the pivotal genes and pathways associated with SIC and shed light on the potential involvement of immune-related ERSRGs. Notably, PDHB was identified as a key driver in SIC, potentially influencing disease progression through the regulation of ERS and metabolism. The diagnostic, prognostic, and therapeutic significance of PDHB in SIC necessitates further exploration in clinical settings.

## Supporting information

S1 Fig Clinical prognosis analysis of other DE-ERSRGs except PDHB. (TIF)

S2 Fig The proportion of neutrophils in SIC according to different algorithms. (TIF)

S3 Fig (TIF)

S1 Table Differential expressed genes. (XLSX)

S2 Table Key module genes. (XLSX)

S3 Table Venn plot. (XLSX)

S4 Table The degree score of 13 DE-ERSRGs. (XLSX)

S5 Table GO enrichment analysis. (XLSX)

S6 Table KEGG enrichment analysis. (XLSX)

S7 Table GSEA results. (XLSX)

S8 Table GSVA results. (XLSX)

S9 Table The proportions of immune cells based on the CIBERSORTx algorithm. (XLSX)

S10 Table The proportions of immune cells based on the MCP algorithm. (XLSX)

S11 Table The proportions of immune cells based on the xCell algorithm. (XLSX)

S1 Raw image (TIF)

## Abbreviations

AUC: area under the curve
DE-ERSRGs: differentially expressed ERS-related genes
DEGs: differentially expressed genes
ERS: endoplasmic reticulum stress
ERSRGs: ERS-related genes
GO: Gene Ontology
GSEA: Gene Set Enrichment Analysis
GSVA: Gene Set Variation Analysis
KEGG: Kyoto Encyclopedia of Genes and Genomes
LASSO: least absolute shrinkage and selection operator
ME: module feature
PDH: Pyruvate dehydrogenase
PPI: protein interaction
qPCR: quantitative real-time polymerase chain reaction analysis
ROC: receiver operating characteristic curve
SIC: Sepsis-induced cardiomyopathy
TF: transcription factor
WGCNA: Weighted gene co-expression network analysis
