# Correction: Role of immune-related endoplasmic reticulum stress genes in sepsis-induced cardiomyopathy: Novel insights from bioinformatics analysis

**DOI:** 10.1371/journal.pone.0338149

**Published:** 2025-12-04

**Authors:** Wan-Jing Zheng, Yan Zhang, Wei-Dong Fu, Xiao-Lei Fu, Xin Yan

The first author’s name is spelled incorrectly. The correct name is: Wan-Jing Zheng. The correct citation is: Zheng W-J, Zhang Y, Fu W-D, Fu X-L, Yan X (2024) Role of immune-related endoplasmic reticulum stress genes in sepsis-induced cardiomyopathy: Novel insights from bioinformatics analysis. PLoS ONE 19(12): e0315582. https://doi.org/10.1371/journal.pone.0315582
