# Supplementary figures and images for: Role of immune-related endoplasmic reticulum stress genes in sepsis-induced cardiomyopathy: Novel insights from bioinformatics analysis
Source: PLoS One. 2024 Dec 13;19(12):e0315582. doi: 10.1371/journal.pone.0315582 (PMC11642931; doi:10.1371/journal.pone.0315582)

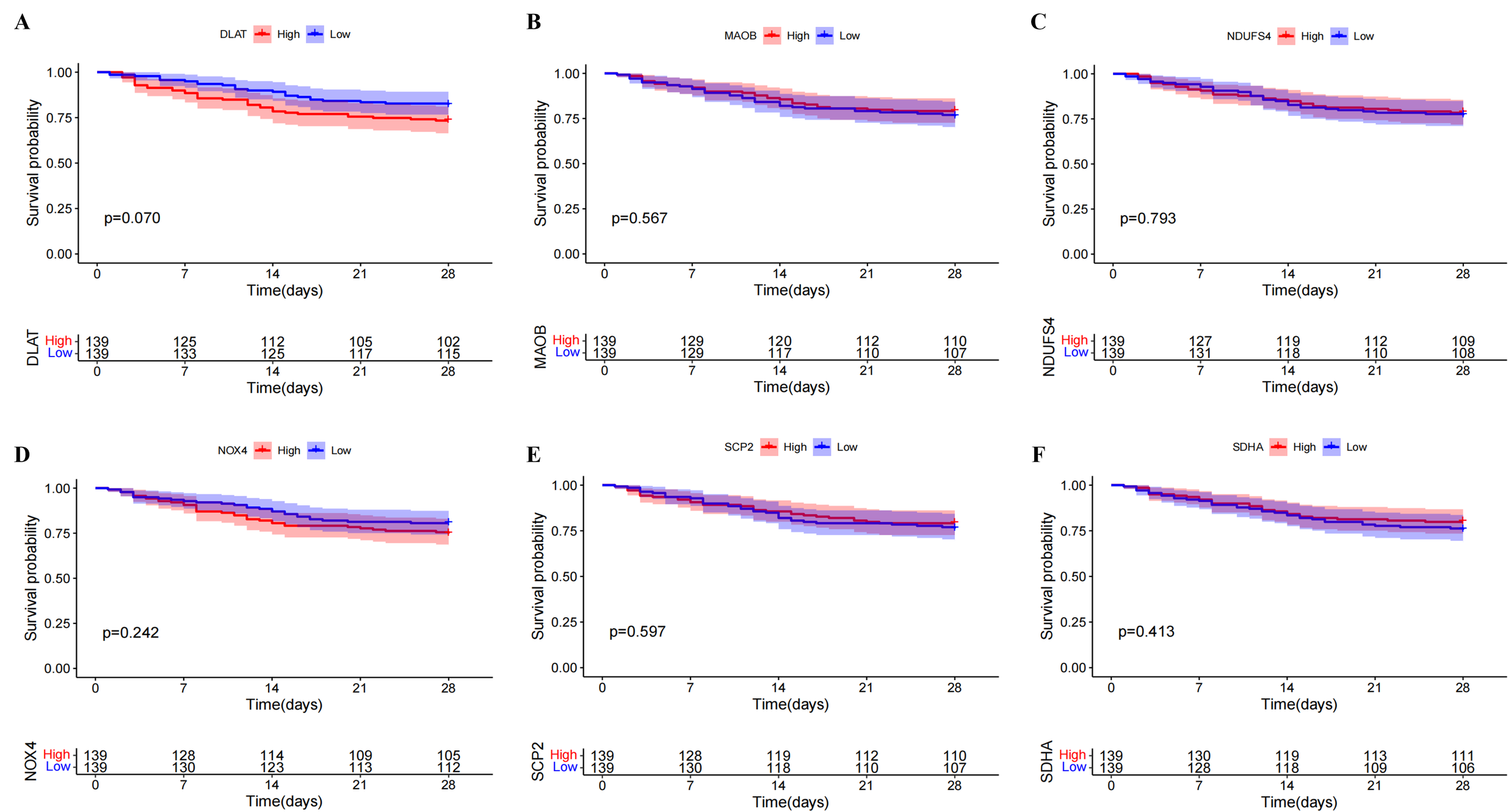

Supplement: S1 Fig — (TIF) [file pone.0315582.s001.tif]

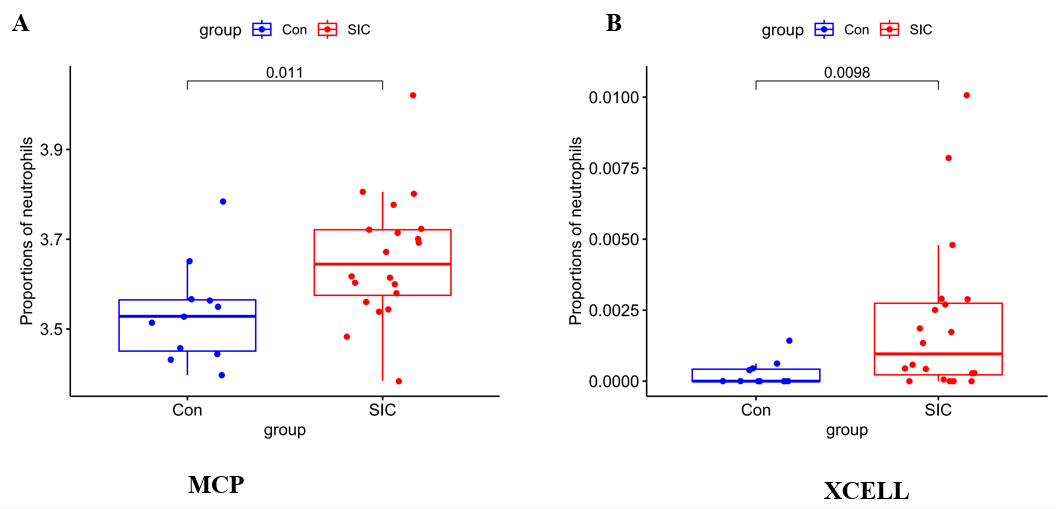

Supplement: S2 Fig — (TIF) [file pone.0315582.s002.tif]

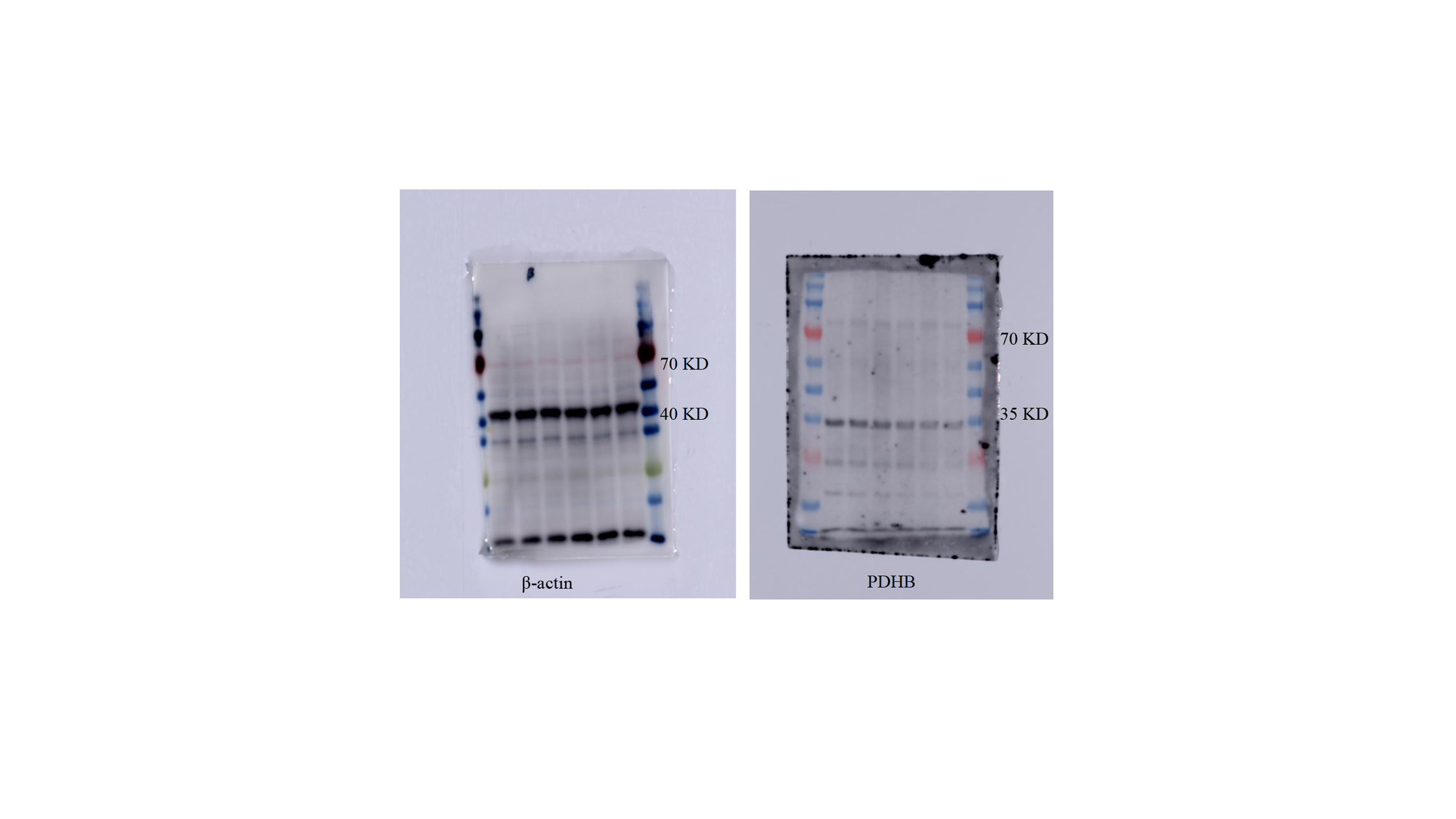

Supplement: S3 Fig — (TIF) [file pone.0315582.s003.tif]

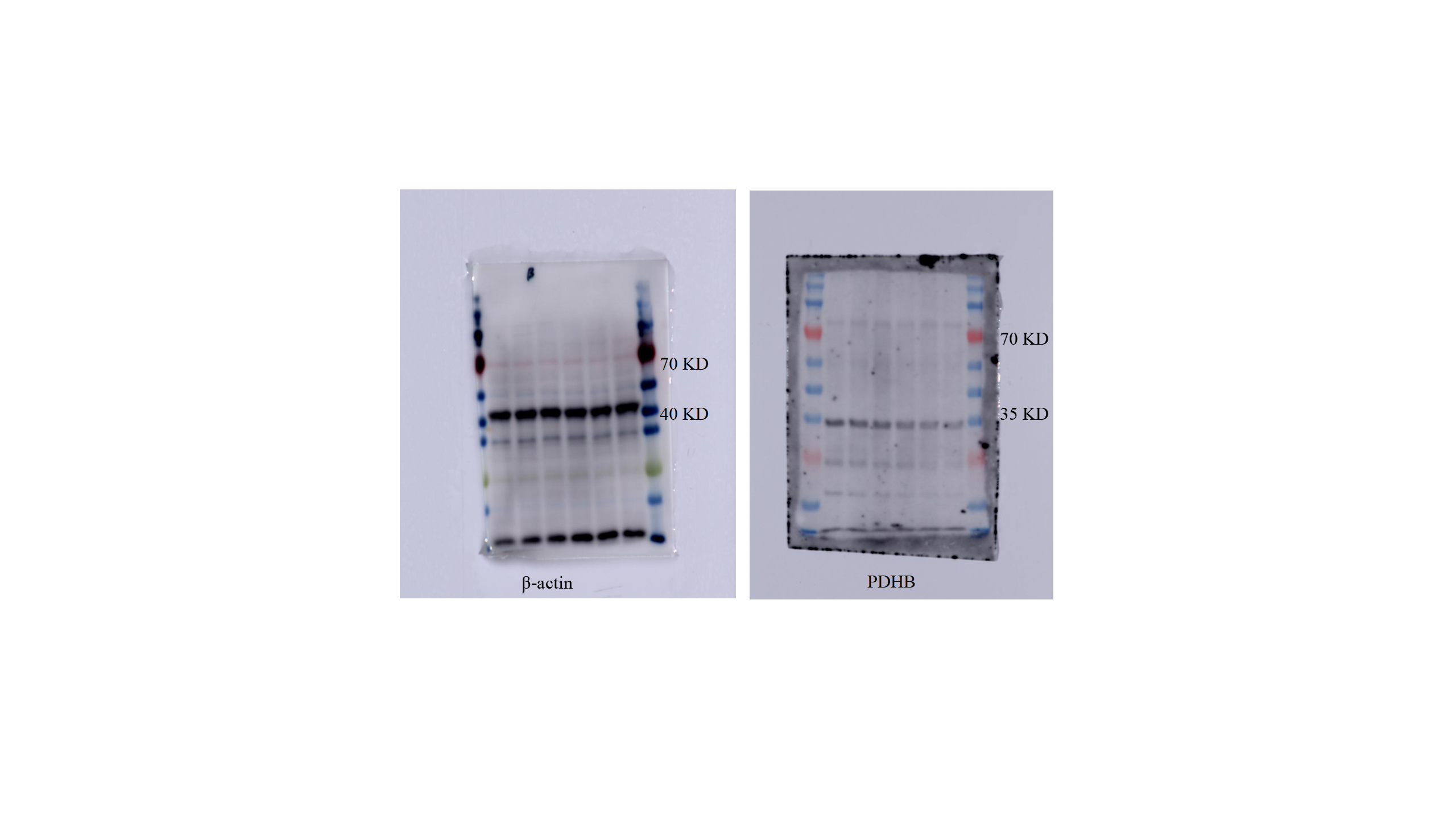

Supplement: S1 Raw image — (TIF) [file pone.0315582.s015.tif]
